# GASAL2: a GPU accelerated sequence alignment library for high-throughput NGS data

**DOI:** 10.1186/s12859-019-3086-9

**Published:** 2019-10-25

**Authors:** Nauman Ahmed, Jonathan Lévy, Shanshan Ren, Hamid Mushtaq, Koen Bertels, Zaid Al-Ars

**Affiliations:** 1 Delft University of Technology, Delft, Netherlands and University of Engineering and Technology, Lahore, Pakistan; 2 Delft University of Technology, Netherlands, Delft, Netherlands; 3 Maastricht UMC+, Netherlands, Maastricht, Netherlands

**Keywords:** Genomics, Sequence alignment, NGS, GPU library

## Abstract

**Background:**

Due the computational complexity of sequence alignment algorithms, various accelerated solutions have been proposed to speedup this analysis. NVBIO is the only available GPU library that accelerates sequence alignment of high-throughput NGS data, but has limited performance. In this article we present *GASAL2*, a GPU library for aligning DNA and RNA sequences that outperforms existing CPU and GPU libraries.

**Results:**

The GASAL2 library provides specialized, accelerated kernels for local, global and all types of semi-global alignment. Pairwise sequence alignment can be performed with and without traceback. GASAL2 outperforms the fastest CPU-optimized SIMD implementations such as SeqAn and Parasail, as well as NVIDIA’s own GPU-based library known as NVBIO. GASAL2 is unique in performing sequence packing on GPU, which is up to 750x faster than NVBIO. Overall on Geforce GTX 1080 Ti GPU, GASAL2 is up to 21x faster than Parasail on a dual socket hyper-threaded Intel Xeon system with 28 cores and up to 13x faster than NVBIO with a query length of up to 300 bases and 100 bases, respectively. GASAL2 alignment functions are asynchronous/non-blocking and allow full overlap of CPU and GPU execution. The paper shows how to use GASAL2 to accelerate BWA-MEM, speeding up the local alignment by 20x, which gives an overall application speedup of 1.3x vs. CPU with up to 12 threads.

**Conclusions:**

The library provides high performance APIs for local, global and semi-global alignment that can be easily integrated into various bioinformatics tools.

## Background

Many applications for processing NGS sequencing data depend heavily on sequence alignment algorithms to identify similarity between the DNA fragments in the datasets. Well known programs for DNA mapping such as BWA-MEM [1] and Bowtie2 [2], DNA assemblers such PCAP [3] and PHRAP [4], make repeated use of these alignment algorithms. Furthermore, in various practical multiple sequence alignment algorithms, many pairwise sequence alignments are performed to align sequences with each other. Also, alignment based read error correction algorithms, like Coral [5] and ECHO [6], perform a large number of pairwise sequence alignments. In addition, variant callers for NGS data e.g. GATK HaplotypeCaller [7], also make use of sequence alignment.

Sequence alignment is the process of editing two or more sequences using gaps and substitutions such that they closely match each other. It is performed using dynamic programming. There are two types of sequence alignment algorithms for biological sequences: *global alignment* and *local alignment*. The former is performed using the Needleman-Wunsch algorithm [8] (NW), while Smith-Waterman algorithm [9] (SW) is used for the latter. Both algorithms have been improved by Gotoh [10] to use affine-gap penalties. These alignment algorithms can be divided into the following classes:

- *Global alignment*: In global alignment, also known as end-to-end alignment, the goal is to align the sequences in their entirety while maximizing the alignment score.
- *Semi-global alignment*: Unlike global alignment, semi-global alignment finds the overlap between the two sequences, allowing to skip the ends of a sequence without penalty. In semi-global alignment the gaps at the leading or trailing edges of the sequences can be ignored, without inducing any score penalty. Different kinds of semi-global alignments are possible depending on which sequence can have its beginning or end be skipped. GASAL2 supports all kinds of semi-global alignments where any combination of beginning or end of a pair of sequences can be ignored.
- *Local alignment*: In local alignment, the goal is to align two sequences so that the alignment score is maximized. As opposed to global alignment, the final alignment may not contain the whole of the sequences. No penalty is induced by misalignments in the beginning and end of the sequences, and the score is kept positive.

**Fig. 1:**
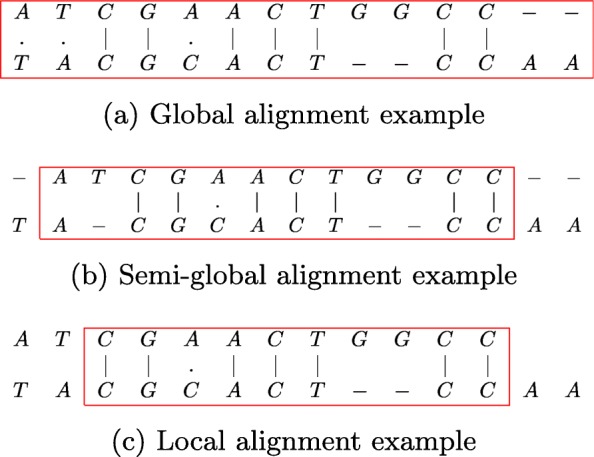
Alignment of *S*_1_ and *S*_2_ sequences shown in Fig. 2. **a** Global alignment example. **b** Semi-global alignment example. **c** Local alignment example

**Fig. 2:**
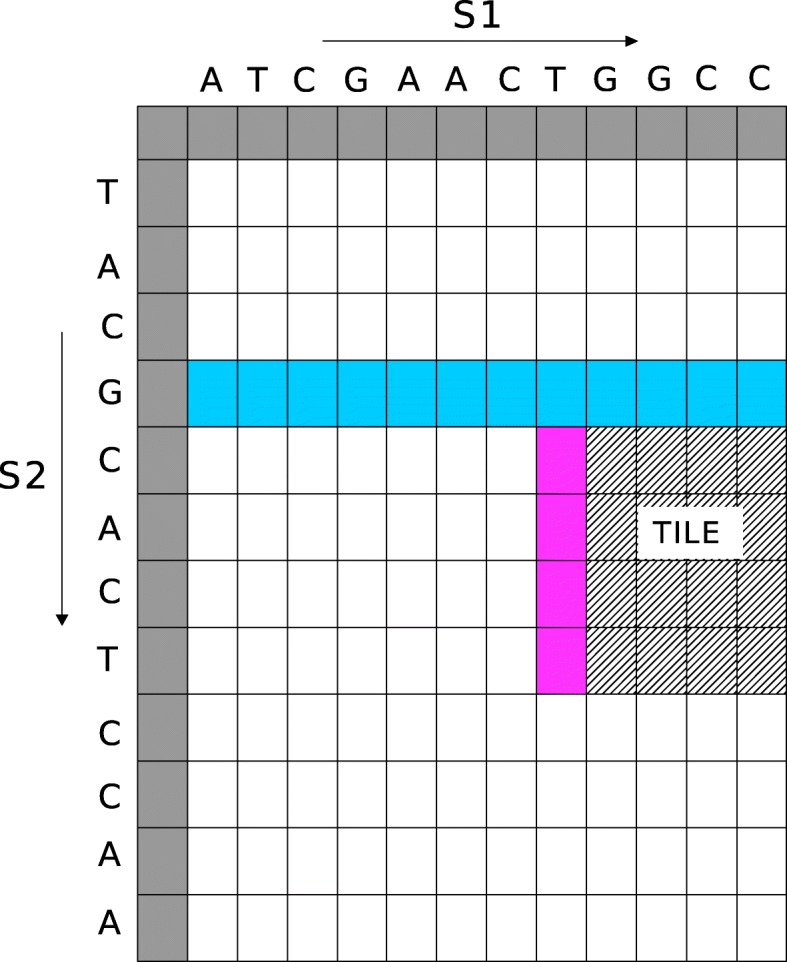
Identical *H*, *E* and *F* matrix

Figure 1 shows the alignment of the two sequences shown in Fig. 2. The bases enclosed in the box constitute the alignment. Match score is 3; mis-match penalty is 4; gap open and gap extension penalties are 6 and 1, respectively. For global alignment the alignment score is -5. In case of semi-global alignment the gaps at the end of *S*_1_ are not penalized. The alignment score is 7, while the start and end positions of the alignment on *S*_2_ are 2 and 10, respectively. For local alignment, the final alignment score is 10. The end-positions of the alignment on *S*_1_ and *S*_2_ are 12 and 10, respectively. The start-position is 3 on both sequences.

### Graphical processing units

Graphical Processing Units (GPUs) were developed for rendering graphics, but are now being used to accelerate many other applications due to their massively parallel architecture. The GPU architecture varies from one vendor to the other and even across different GPU generations from the same vendor. Here we give a general overview of state-of-the-art NVIDIA GPUs. The cores of a GPU, known as streaming processors (SPs), groups of which are organized into a number of streaming multiprocessors (SMs). Each SM has a set of SPs, a register file, one or more thread schedulers, a read only memory, L1 cache, shared memory, and some other hardware units. All SMs access the DRAM (known as global memory) through a shared L2 cache. The programming language for NVIDIA GPUs is known as *CUDA* which is an extension of C/C++. The function that executes on the GPU is known as *kernel*. The data to be processed by the kernel is first copied from the CPU memory into the global memory of the GPU. The CPU (known as the *host*) then launches the kernel. Once the kernel is finished the results are copied from the global memory back into CPU memory. This copying of data back and forth between host and GPU is quite time expensive. Therefore, data is transferred between the host and GPU in the form of large batches to keep number of transfers at minimum. Moreover, the batch should be large enough to fully utilize the GPU resources.

At every clock cycle each SM executes instructions from a group of threads known as a *warp*. A *warp* is a set of 32 GPU threads that execute in lock-step (i.e., they share the instruction pointer). Therefore, if one or more threads execute a different instruction, different execution paths are serialized causing performance loss. This phenomenon is known as *divergent execution* and should be avoided as much as possible. Moreover, to achieve good memory throughput the memory accesses should be coalesced (i.e., all the threads in a warp should access consecutive memory locations).

To allow the overlapping of GPU and CPU execution, all the GPU kernel launches are asynchronous i.e. control is immediately returned to the CPU after the kernel launch. In this way, the launching thread can perform other tasks instead of waiting for the kernel to complete. Using *CUDA streams*, it is possible to launch one or more kernels on GPU before the results of a previously launched kernel has been copied back to the CPU. CUDA streams also allow to asynchronously perform the copying operations. Hence, one can just launch all the operations and perform other tasks on the CPU. Subsequently, the cudaStreamQuery() API function can be used to test whether all the operations in a given stream have completed or not.

### Previous research works

GPU acceleration of sequence alignment has been the topic of many research papers like [11–13]. Apart from sequence alignment, GPUs are also used for accelerating many other bioinformatics algorithms, such as, described in [14, 15]. Moreover, various biomedical image analysis applications are accelerated with GPUs. Kalaiselvi et al. [16] surveys the GPU acceleration of medical image analysis algorithms. In [17, 18], GPUs are used to accelerate the processing of MRI images for brain tumour detection and segmentation. Most of the previous work on accelerating sequence alignment, was focused on developing search engines for databases of protein sequences. The alignment of DNA and RNA sequences during the processing of high-throughput NGS data poses a different set of challenges than database searching as described below.

1. The sequences to be aligned in NGS processing are generated specifically for each experiment. In contrast, in database searching, the database of sequences is known in advance and may be preprocessed for higher performance.
2. In database search programs, one or few query sequences are aligned against all the sequences in the database (may be regarded as target sequences), whereas the processing of NGS data requires pairwise one-to-one, one-to-many or all-to-all pairwise sequence alignment. Due to this, a common performance improvement technique in database search programs, like using *query profile*, is not feasible in NGS data alignment.
3. In programs containing GPU accelerated sequence alignment, the alignment step is tightly coupled with the rest of the program. The GPU alignment kernel is specifically tailored to meet the requirements of the program. Therefore, reusing the kernel to accelerate the sequence alignment in other programs is not easy.

Due to these differences, GPU accelerated database search cannot be used to accelerate the alignment step in NGS data processing programs. gpu-pairAlign [19] and GSWABE [20] present only all-to-all pairwise local alignment of sequences. All-to-all alignment is easier to accelerate on GPU. Since, only one query sequence is being aligned to all other sequences, the query sequence may reside in the GPU cache, substantially reducing global memory accesses. On the other hand, in one-to-one alignment each query sequence is different limiting the effectiveness of caching these sequences. In many NGS data processing applications, one-to-one pairwise alignment is required (e.g., DNA read mapping). In DNA read mapping, local alignment takes a substantial percentage of the total run time. For example, in the BWA-MEM DNA read aligner the local alignment takes about 30% of the total execution time with query lengths of 250bp (or base pairs), while calculating only the score, start-position and end-position.

None of the previously published research efforts have developed any GPU accelerated sequence alignment library that can be easily integrated in other programs that require to perform pairwise alignments. NVBIO [21] is the only public library that contains GPU accelerated functions for the analysis of DNA sequences. Although this library contains a GPU accelerated function for sequence alignments, its performance is limited. Therefore, in this paper we present a GPU accelerated library for pairwise alignment of DNA and RNA sequences, GASAL2 (GPU Accelerated Sequence Alignment Library v2), as an extension of our previously developed GASAL library described in [22]. The library contains functions that enable fast alignment of sequences and can be easily integrated in other programs developed for NGS data analysis. Functions for all three types of alignment algorithms (i.e., local, global and semi-global) are available in GASAL2. One-to-one as well as all-to-all and one-to-many pairwise alignments can be performed using affine-gap penalties. The contributions of the paper are as follows:

- A GPU accelerated DNA/RNA sequence alignment library that can perform global, semi-global (all types) as well as local alignment between pair of sequences. The library can compute the alignment score and the actual alignment between two sequences by performing traceback. The actual alignment is generated in CIGAR format and contains the exact position of matches, mismatches, insertion and deletion in the alignment. Optionally it can compute the alignment score with only the end, and if required, the start position of the alignment.
- The library uses CUDA streams so that the alignment functions can be called asynchronously and the host CPU can perform other tasks instead of waiting for the alignment to complete on the GPU.
- GASAL2 is the fastest sequence alignment library for high-throughput Illumina DNA sequence reads in comparison to highly optimized CPU-based libraries, and it is also much faster than NVBIO, NVIDIA’s own GPU library for sequence analysis.
- GASAL2 can be easily integrated in bioinformatics applications, such as accelerating the seed-extension stage of BWA-MEM read mapper.

## Implementation

In this paper, we describe GASAL2, a GPU accelerated library for pairwise sequence alignment. The sequences are first transferred to the GPU memory, where they are *packed* into unsigned 32-bit integers. If needed, any number of sequences can then be reverse-complemented. Finally, the alignment is performed and the results are fetched back from the GPU memory to the CPU memory. This section gives an overview of the implementation choices of GASAL2 and describes various stages in the data processing pipeline performed on the GPU.

### Stage-1: data packing

**Fig. 3:**
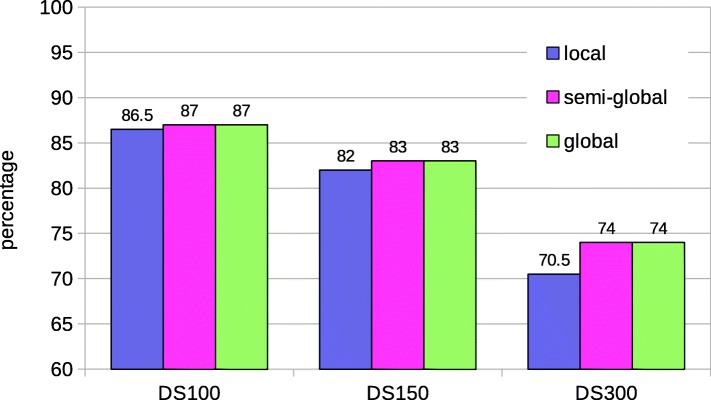
NVBIO data packing time as percentage of total execution time

**Fig. 4:**
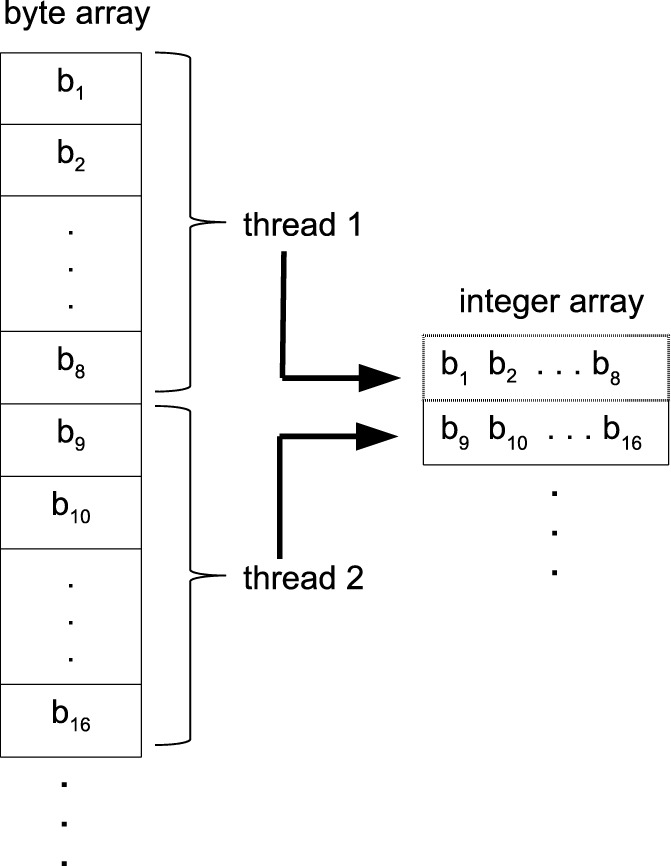
Packing the sequences on GPU. *b*_1_,*b*_2_,…, are the bases

**Fig. 5:**
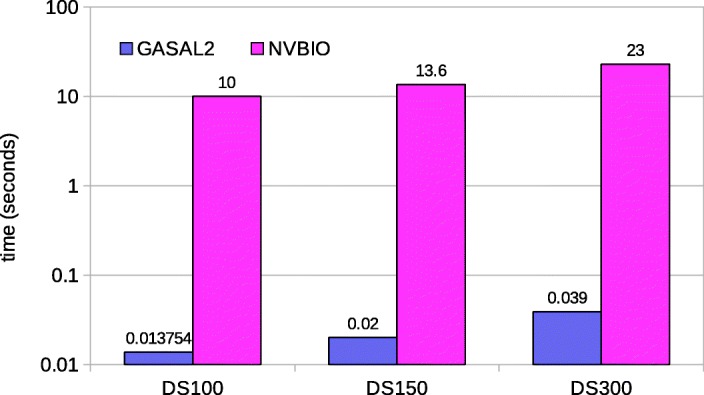
Data packing time, GASAL2 vs NVBIO

The user passes the two batches of sequences to be pairwise aligned. A batch is a concatenation of the sequences. Each base is represented in a byte (8-bits). DNA and RNA sequences are made up of only 5 nucleotide bases, A, C, G, T/U (T in case of DNA and U in RNA) and N (unknown base), 3 bits are enough to represent each symbol of a sequence. However, we represent each base in 4 bits for faster packing. Due to the compute bound nature of the GASAL2 alignment kernel, using 3-bits does not result in any significant speedup over the 4-bit representation, but instead complicates the data packing process. Registers in the GPU are 32-bits wide. Therefore, a batch of sequences is packed in an array of 32-bit unsigned integers in which each base is represented by 4 bits. NVBIO also packs the sequences on CPU using 4 bits per base. As the total number of bases in a batch is quite large, packing the data on the CPU is very slow. Figure 3 shows the percentage of data packing in the total execution time for one-to-one pairwise alignment of the input dataset. The input dataset and GPU platform are described in “Input dataset and execution platforms” section on page 6. Figure 3 shows that NVBIO data packing takes around 80% of the total time. Hence, in NVBIO preparing the sequences for the alignment on GPU takes much more time then actually aligning the sequences. Based on this observation, we accelerate the data packing process on GPU and unpacked batches of sequences are copied to the GPU global memory for this purpose. Figure 4 shows how the GPU data packing kernel works on GPU. Each GPU thread loads eight bases at a time from global memory. Each base is converted from 8-bit to 4-bit representation by masking the upper 4 bits, and then packed into an unsigned 32-bit integer which is written back to global memory. Figure 5 shows the achieved speedup of our novel approach of packing the sequences on GPU as compared to sequence packing performed by NVBIO on CPU. GASAL2 is at least 580x faster than NVBIO. Since, only few milliseconds are required to pack the sequences in GASAL2, the data packing time is completely eliminated. After the data packing is complete, packed sequences reside on the GPU memory and all subsequent operations are completely done on the GPU, only the final results of the alignment need to be copied from GPU to CPU.

### Stage-2 (optional): reverse-complementing kernel

GASAL2 is able to reverse and/or complement any number of sequences from any batch. Any sequence can be flagged to be reversed, complemented, or reverse-complemented. The reverse-complementing process is performed on the GPU on already packed sequences to take advantage of the high parallelism of the task.

### Stage-3: alignment

The sequence alignment kernel is launched to perform pairwise alignment of the sequences using affine-gap scoring scheme. GASAL2 employs inter-sequence parallelization and each GPU thread is assigned a pair of sequences to be aligned. All pairs of sequences are independent of the others, so there is no data dependency and all the alignments run in parallel. An alignment algorithm using affine-gap penalties compute cells in three dynamic programming (DP) matrices. These matrices are usually named as *H*, *E* and *F*. The matrices are shown in Fig. 2. Each cell needs the results of 3 other cells: the one on top, the one on the left, and the one on the top-left diagonal. Since the sequences are packed into 32-bits words of 8 bases each, the alignment fetches a word of both sequences from memory and computes an 8x8 tile of the matrix. Hence, 64 cells of the DP matrices are computed with a single memory fetch reducing the number of memory requests. All the tiles are computed from left to right, then top to bottom. To jump from one tile to the next one on the right, we need to store 8 intermediate values (which are the values of the cell of the left for the next tile). To jump from one row of tiles to the next row, we need to store a full row of intermediate values (which are the values of the cell of the top for the next row of tiles). Hence, instead of storing the whole matrix, we only store an 8-element column and a full row, which reduces the memory requirement from *O*(*n*^2^) to *O*(*n*). Since, the stored column has only 8 elements it can easily reside in the GPU register file. For ease of representation, Fig. 2 shows a 4 x 4 tile, and the intermediate values that are stored are shown shaded. Our library can also compute the start-position of the alignment without computing the traceback. To do so, we restart the computation, but now from the end-position in the backward direction, and exit where the score becomes equal to the previously found score. The coordinates of the cells at the exit point give the start-position of the alignment.

For computing the traceback a *direction* matrix is stored in the global memory of the GPU while computing the alignment. The direction matrix is similar to the one shown in Fig. 2 with |*S*_1_|×|*S*_2_| cells. Each cell is represented by 4-bits in the memory. The lower 2 bits are used to encode whether the current cell is match, mismatch, insertion or deletion. The upper two bits are for the next cell on the alignment path. If the next cell is a gap then the upper bits of the current cell represent whether it is a gap-open or gap-extension, one bit each for insertion and deletion. The direction matrix is stored in the memory using uint4 CUDA vector data type. uint4 has 4 aligned 32-bit unsigned integers. A single store/load instruction is required to access uint4 data from the memory. A single uint4 data element can store 32 direction matrix cells, and hence half the cells in a tile. Moreover, the direction matrices of all the pairs aligned on the GPU are stored in an interleaved fashion for coalesced memory access. The actual alignment is generated using the direction matrix by starting from the end cell and tracing back to the start of the alignment to compute the exact location of matches, mismatches, deletions and insertions.

The output of this stage depends on the users choice. There are three possible outputs: 1) only score and end-position of the alignment. 2) score, end-position and start-position of the alignment without performing traceback. 3) score, end-position, start-position and actual alignment in CIGAR format.

### Kernel specialization through templates

GASAL2 supports various kinds of parameters for kernel launches, to tailor the results to the user’s need. For example, the traceback will only be calculated if the user requests it. In addition, GASAL2 can adapt to any kind of semi-global alignment where the initialization or the search for a maximum can vary, depending on the user requesting the beginning and/or the end of any sequence.

Dealing with this kind of issue is not trivial in the case of GPU programming, as creating a simple branch through an *if* statement slows down the whole kernel dramatically (for a single *if* in the innermost loop of an alignment kernel, this can cause an approximate slowdown of 40%). Duplicating the kernels is not a viable solution for code maintenance: for example, for the semi-global kernel, there are 2^4^=16 types; and adding the possibility of asking for the start-position doubles this number.

The solution that we adopted allows to generate all the possible kernels at compilation time, so that they are all ready to run at full speed without branches. CUDA implementation of C++ templates (according to C++11 specifications) allows to generate all template-kernels at compile time. The programming model that we adopted allows to create a new kernel specialization by writing *if* statements that are resolved at compilation time, to prune the useless branches.

### GPU launch parameters choice

GPU threads are organized in *blocks*, and blocks are grouped into *kernel grid*. A *block* is run on a SM that has several hardware resources such as cores, register file, cache, etc. Two parameters characterize the kernel launch:

- the block size, which is the number of threads in a block.
- the grid size, which is the total number of blocks.

Block size affects the *SM occupancy*. The SM occupancy is the ratio of number of active warps and the maximum number of warps allowed on a SM. Increasing the occupancy helps in memory-bound applications. Large occupancy makes sure that they are always enough number of warps that are ready to be scheduled to the streaming processors so that all cores (SP’s) in the SM are fully utilized. GASAL2 alignment kernel is not memory-bound. It can compute a 8x8 tile of cells in only 2-3 memory requests. Thus, increasing the occupancy does not help much. However, GASAL2 alignment kernels use a block size of 128 for reasonable occupancy value. GASAL2 uses the inter-sequence parallelization and each GPU thread performs only one alignment. Hence, the grid size is always the ratio of number of alignments to be performed and the block size (128).

### GASAL2 asynchronous execution

GASAL2 allows the user to overlap GPU and CPU execution. This is known as *asynchronous* or *non-blocking* alignment function call as opposed to *synchronous* or *blocking* call used in GASAL [22]. In a blocking alignment function call, the calling thread is blocked until the alignment on the GPU is complete. GASAL2 uses CUDA streams to enable asynchronous execution. In asynchronous calls, the calling thread is not blocked and immediately returns after launching various tasks on the GPU. In GASAL2 these tasks are CPU-GPU memory transfers, and the GPU kernels for data packing, reverse-complementing (optional), and pairwise-alignment. The application can perform other tasks on the CPU rather than waiting for the GPU tasks to complete. This helps to eliminate idle CPU cycles in case of a blocking call. Hence, the time spent in the alignment function is merely a small overhead to call the CUDA API asynchronous memory copy functions and launch the kernels.

### GASAL2 versus GASAL and NVBIO

The advantages of GASAL2 over GASAL and NVBIO are listed below:

1. GASAL2 can generate the actual alignment between a pair of sequences by computing traceback. The traceback contains the exact position of matches, mismatches, insertion and deletion in the alignment. This facility is not provided in GASAL.
2. GASAL2 is much faster than NVBIO.
3. Asynchronous execution. This is a unique facility that is not available in NVBIO or GASAL.
4. In NVBIO and GASAL, an ambiguous base (N) is treated as a ordinary base having the same match and mismatch scores as A, C, G or T. But, in most sequence analysis programs, the match/mismatch score of "N" is different. For example, in BWA-MEM the score of aligning "N" against any other base (A, C, G, T or N) is always -1. Extending NVBIO to adopt this new scoring scheme to handle "N" increases the execution time of GPU kernels by 30% for global and semi-global alignment, and by 38% for local alignment. In GASAL2 the score of aligning "N" against any other base is configurable. Due to this, the execution time of global, semi-global and local kernels is higher than that of GASAL by 17%, 15% and 6%, respectively.
5. In GASAL, the GPU memory allocations are performed just before the batch of sequences are copied from CPU to GPU. The allocated memory is freed after the alignment is complete and the results are copied from GPU to CPU. If the input batch is not very large, the time spent in memory allocation and de-allocations may become significant and, thus reduces the performance. In GASAL2, we have a separate API function for memory allocation and de-allocation which is called only once at the beginning and end of the program, respectively. At the beginning of the program, user calls the memory allocation function by passing an estimated input batch size. Separate data structures are maintained to keep track of the allocated memory. If the actual input batch is larger, GASAL2 automatically handles the situation by seamlessly allocating more memory. The allocated memory is freed up at the end of the application.
6. GASAL2 supports all types of semi-global alignments. NVBIO and GASAL supports only one type of semi-global alignment in which the gaps at the beginning and end of the query sequence are ignored.
7. GASAL2 can also compute the second-best local alignment score. GASAL only computes the best score.
8. GASAL2 has a reverse-complementing GPU kernel. In NVBIO and GASAL, the user has to manually reverse-complement the sequence before writing it to the input batch.

## Results

### Input dataset and execution platforms

To evaluate the performance of GASAL2 we performed *one-to-one pairwise* alignments between two set of sequences. We considered the case of DNA read mapping. Read mappers have to perform billions of one-to-one pairwise alignments between short segments of DNA and substrings of the reference genome. In this paper, we also perform one-to-one pairwise alignments between two set of sequences for evaluation purposes. Affine-gap scoring scheme is used in which the match score, mis-match penalty, gap open penalty and gap extension penalty is 6, 4, 11 and 1, respectively. In the rest of the paper,we will refer to the substrings of the reference genome as *target* sequences. The length of the read sequence is fixed, while the length of the target sequence may vary. Table 1 shows the different datasets used in this paper. The read set consists of reads simulated with Wgsim [23] using UCSC hg19 as the reference genome. To generate the target set, these reads and the hg19 reference genome are used as the input for BWA-MEM. During the seed-extension phase of BWA-MEM, the mapper aligns the reads with the substrings of the reference genome. These substrings are stored and used as the target set. Three typical read lengths generated by Illumina high-throughput DNA sequencing machines are used: DS100, DS150 and DS300 representing 100, 150 and 300bp, respectively. Table 1 shows the number of sequences in the read and target set and the corresponding maximum and average length of the sequences in each set. Minimum target sequence length in each case is approximately equal to the length of the read.

**Table 1:** Characteristics of the input dataset

Dataset	Read Set			Target Set		
	avg. len.	max. len.	No. of seq.	avg. len.	max. len.	No. of seq.
DS100	100	100	10e6	162	177	10e6
DS150	150	150	10e6	260	277	10e6
DS300	300	300	10e6	538	571	10e6

The CPU-based libraries are executed on a high end machine consisting of two 2.4 GHz Intel Xeon E5-2680 v4 (Broadwell) processors and 192 gigabytes of RAM. Each processor has 14 two-way hyper-threaded cores. Hence, there are 28 physical and 56 logical cores in total. We measured the execution time of the CPU-based libraries with 28 and 56 threads and reported the smallest execution time of the two. GASAL2 and NVBIO are executed on a NVIDIA Geforce GTX 1080 Ti GPU. Only one CPU thread is used in case of GASAL2 and NVBIO. GASAL2 is compiled with CUDA version 10.0.

### Libraries compared with GASAL2

We compared GASAL2 against the fastest CPU and GPU based libraries available, which are:

- *SeqAn* contains the vectorized implementation of all types of alignments using SSE4, AVX2 and AVX512 SIMD instructions [24]. For SeqAn we used the test-suite provided by the developers of the library [25]. AVX2 implementation of SeqAn is used in the experiments with 16 bits per score. Since the test data set is based on Illumina reads, we have used align_bench_par and align_bench_par_trace which follows the *chunked* execution policy giving the fastest execution for short DNA reads. The chunked policy is also used to generate the results in [24] for Illumina reads. align_bench_par calculates the alignment score and does not report the start and end positions of the alignment. We have not used the banded version of align_bench_par as it does not guarantee correct results. align_bench_par_trace is used for computing alignment with traceback. In this paper, we are performing one-to-one alignment for the experiments. The timings reported in the SeqAn paper [24] are not for the one-to-one alignment. The paper used a so-called "olc" alignment mode which is similar to the different one-to-many alignments. The library is compiled with GCC 7.3.1.
- *ksw* module in klib [26] contains a fast SSE based implementation local alignment algorithm. It can also compute the start-position, but does not compute the traceback for local alignment. It has a function for computing the traceback for global alignment, but it is not vectorized, and hence very slow. ksw is faster than SSW [27]. We developed our own test program for ksw (commit:cc7e69f) which uses OpenMP to distribute the alignment tasks among the CPU threads. The test program is compiled with GCC 4.8.5 using O3 optimization flag.
- *Parasail* [28] contains the SIMD implementation of the local, global and semi-global alignment with and without traceback. Ten types of semi-global alignments are supported. We developed our own test program for Parasail (version-2.4) which uses OpenMP to distribute the alignment tasks among the CPU threads. The test program is compiled with GCC 4.8.5 using O3 optimization flag. Parasail allows the user to choose between SSE and AVX2 SIMD implementations. It also consists of different vectorization approaches namely *scan*, *striped*, *diagonal* and *blocked*. We have used the *scan* approach implemented with AVX2 instructions as it is the fastest for our dataset. Parasail does not compute the start-position directly without computing traceback. Therefore, the original sequences are aligned to obtain score and end-position, then both sequences are reversed to calculate the start-position without traceback.
- *NVBIO* contains the GPU implementations of local global and semi-global alignment with and without traceback. Only one type of semi-global alignment is supported shown in Fig. 1. We used sw-benchmark program in the NVBIO repository. The original program performs one-to-all alignments. We modified sw-benchmark to perform one-to-one alignments. Moreover, in the original program reading the sequences from the files and packing the sequences is done in a single API function call. To exclude the I/O time from the measurements, we first loaded the sequences in an array of strings and then pack the sequences using NVBIO API functions. NVBIO does not contain any function that directly computes the start-position of the alignment without computing the traceback. To compute the start-position without traceback, we make two copies of each sequence, one in original form and other reversed. The alignment of original sequences is used to compute the score and end-position, while the reverse sequence are aligned to compute the start-position. Moreover, as described before, NVBIO considers "N" as an ordinary base and extending the library to correctly handle the ambiguous base makes it more than 30% slower. In our comparison we have used the original NVBIO implementation. NVBIO is compiled with CUDA version 8 as it cannot be compiled with latest CUDA version.

There are also very fast CPU-based libraries that compute the edit distance or sequence alignment with linear-gap penalty e.g. EDlib [29], BitPAl [30] and [31]. EDlib computes the Levenshtein distance between two sequences. Edit distance is the minimum number of substitution, insertions and deletion required to transform one sequence to the other. BitPAl and BGSA [31] can perform global and semi-global alignments with linear-gap penalty. Many bioinformatics applications require sequence alignment with affine-gap penalty which allows to have different penalties for gap opening and gap extension. Moreover EDlib, BitPAl and BGSA cannot compute local alignment.

### GASAL2 alignment kernel performance

Table 2 shows a comparison of the alignment kernel execution times of NVBIO and GASAL2. The times listed in the table represent the total time spent in the GPU alignment kernel while performing all the one-to-one pairwise alignment between the sequences in the read and target set. These times do not include data packing and data copying time. Three different types of kernels are timed. The “only score” kernels only compute the score and end position. The “with start” kernels compute the score as well as start and end position without computing the traceback. There is no need to compute the start position for global alignment. The “with traceback” computes the actual alignment along with the score, start-position and end-position. The table shows that the alignment kernel execution times of NVBIO and GASAL2 are almost the same with and without computing the start-position. For finding the start-position GASAL2 kernel first finds the score and end-position. It then again aligns the two sequences in the backward direction beginning form the cell corresponding to the end-position. This backward alignment is halted as soon as its score reaches the previously calculated maximum score. This approach helps to reduce the number of DP cells need to be computed for finding the start-position. With traceback computation GASAL2 GPU kernels are around 4x faster than NVBIO. On the other hand, NVBIO is more space efficient and uses an approach similar to Myers-Miller algorithm [32] to compute the traceback.

**Table 2:** Alignment kernel times (in seconds) for NVBIO and GASAL2

	DS100		DS150		DS300	
GPU kernel	NVBIO	GASAL2	NVBIO	GASAL2	NVBIO	GASAL2
Local (only score)	1	1	2.2	2.2	8.4	9.6
Local with start	2	1.9	4.4	3.3	16.8	13.6
Local with traceback	6	1.58	14	3.6	57.8	15.5
Semi-global (only score)	0.9	1	2	2.2	8	9.3
Semi-global with start	1.8	1.8	4	3.9	16	16
Semi-global with traceback	6	1.43	14	3.4	62	15
Global (only score)	0.9	1	2	2.3	8	9.5
Global with traceback	6	1.4	14	3.5	63	15

### Total execution time

**Fig. 6:**
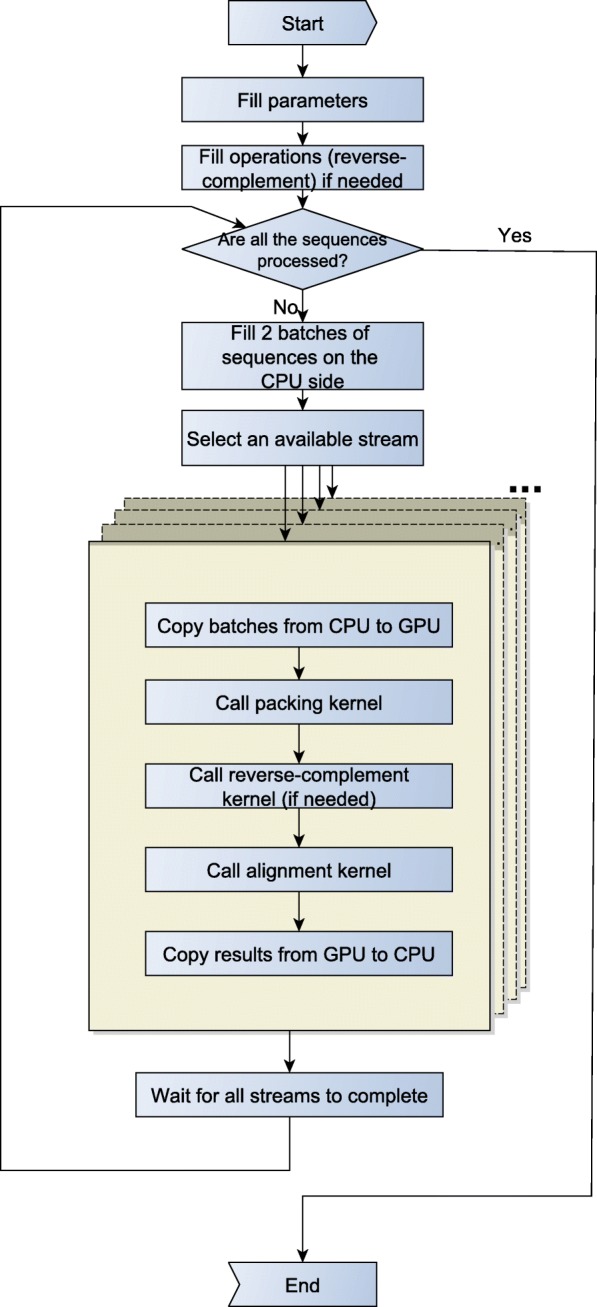
The flow chart of the test program used to measure the total execution time of GASAL2

In this section, we compare the performance of GASAL2 and other libraries in terms of the total execution time. The total execution time is the total time required to perform all the one-to-one pairwise alignment between the sequences in the read and target set. Figure 6 shows the flow chart of the test program used to measure the total execution time of the GASAL2. While filling the parameters we specify the type of alignment algorithm and one of the three following types of computations: 1) only score and end-position. 2) score, start and end-position without traceback. 3)score, end-position start-position and actual alignment in CIGAR format. Two batches of 500K sequences each are filled in each iteration. Hence, there are 20 iterations in total for the dataset of 10 million pair of sequences. GASAL2 initializes 5 CUDA streams and each stream performs one-to-one alignment of 100K pair of sequences. The total execution time of GASAL2 is the time starting from selecting an available stream till the time all the streams are completed i.e. allowing all the operations, from copying batches to copying results, to finish. Since the data transfer time is much smaller than the GPU alignment kernel time (at most 30% of kernel time) and GASAL2 uses CUDA streams, the data transfer is almost entirely overlapped with GPU execution. For the experiments, we are not reverse-complementing the sequences.

#### Local alignment

**Fig. 7:**
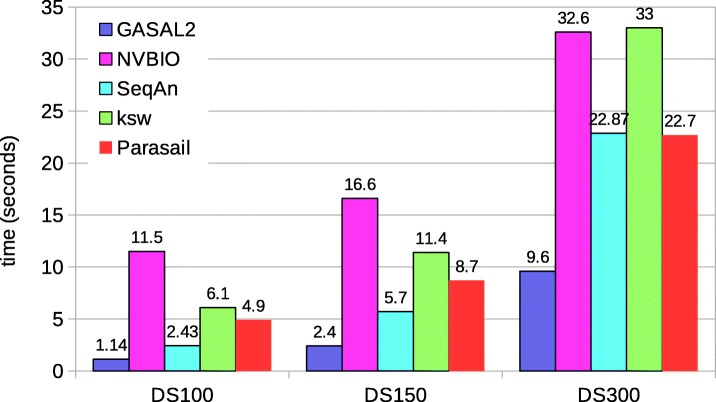
Total execution times for local alignment computing only the score and end-position. The execution time of CPU-based libraries is obtained with 56 threads

Figure 7 shows the total execution times computing only the score and end-position of the alignment. In this case GASAL2, NVBIO, ksw and Parasail are reporting the score as well as the end-position of the alignment. SeqAn only reports the alignment score. The execution times for SeqAn, ksw and Parasail shown in Fig. 7 are obtained with 56 CPU threads. For DS100, the figure shows that GASAL2 is 5.35x, 4.3x, 10x and 2x faster than ksw, Parasail, NVBIO and SeqAn, respectively. With DS150 the speedup of GASAL2 over ksw, Parasail, NVBIO and SeqAn is 4.75x, 3.6x, 7x and 2.4x, respectively. GASAL2 is 3.4x, 2.3x, 3.4x and 2.4x faster than ksw, Parasail, NVBIO and SeqAn, respectively for DS300. These results indicate that the speedup achieved by GASAL2 over ksw and Parasail decreases with longer reads. This is due to the fact that the ksw and Parasail use the striped heuristic that limits the computational complexity for longer reads, as compared to the GPU implementation. The results also show that the speedup achieved by GASAL2 compared to NVBIO decreases with longer reads. The reason for this decreasing speedup over NVBIO with increasing read lengths is the reduction of the data packing percentage (Fig. 3) as the alignment time continues to increase. GASAL2 speeds up the data packing while its alignment kernel performance remains similar to that of NVBIO. The speedup of GASAL2 over SeqAn remains constant around 2x with increasing read lengths. This is because both of them employ inter-sequence parallelization and use the standard DP algorithm having the complexity of |*S*1|×|*S*2| (Fig. 2). Hence, the execution time increases quadratically with read length for both GASAL2 and SeqAn.

**Fig. 8:**
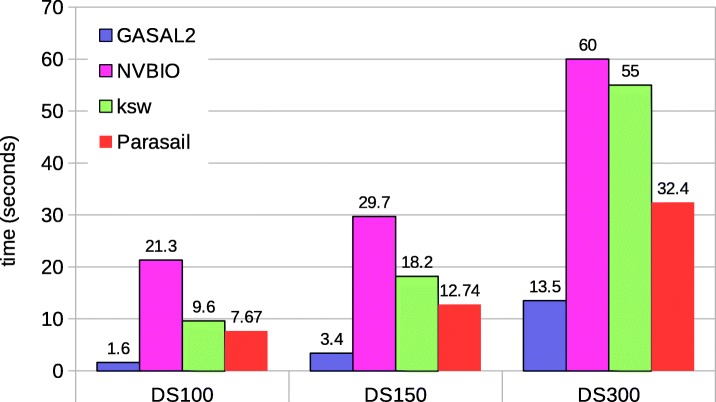
Total execution times for local alignment computing start-position without traceback. The execution time of CPU-based libraries is obtained with 56 threads

Figure 8 shows the total execution time computing the start-position of the alignment without traceback. Since SeqAn neither reports the end-position nor the start-position, it is omitted in this comparison. The execution time values shown for ksw and Parasail are obtained with 56 CPU threads. The figure shows that GASAL2 is 6x, 5.3x and 4x faster than ksw; 4.8x, 3.7x and 2.4x faster than Prasail; 13x, 8.7x and 4.4x faster than NVBIO for DS100, DS150 and DS300 respectively. The reason for decreasing speedup of GASAL2 over CPU-based libraries is the same as described for local alignment without computing the start-position. The speedup over NVBIO is more in this case as compared to alignment without start-position computation. With start-position computation the packing time of NVBIO nearly doubles but the packing time of GASAL2 remains the same. Another interesting point to note is that the GASAL2 total execution time with start-position computation is smaller than the total alignment kernel time shown in Table 2. This happens because the alignment kernels of 5 batches are launched in parallel and their execution may overlap on GPU.

**Fig. 9:**
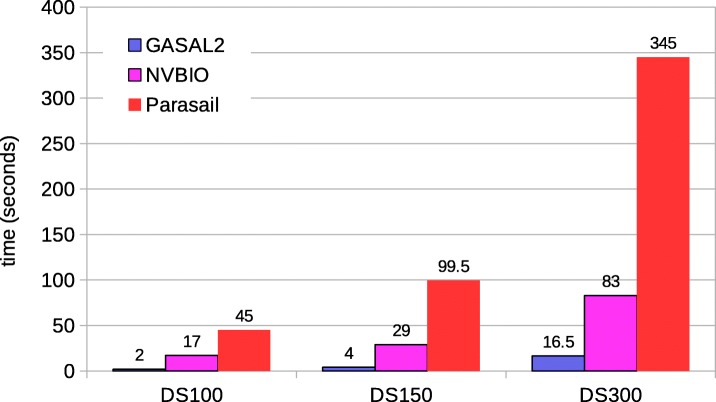
Total execution times for local alignment with traceback computation. The execution time of CPU-based libraries is obtained with 56 threads

Figure 9 shows the total execution of the local alignment with traceback. The traceback computation gives the actual alignment between the pair of sequences along with the score, end-position and start-position. SeqAn and Parasail timings are obtained with 56 CPU threads. GASAL2 is 8.5x, 7.25x and 5x faster than NVBIO for DS100, DS150 and DS300, respectively. With increasing read lengths the data packing percentage in NVBIO decreases but the kernel speedup of GASAL2 over NVBIO remains constant (4x). The speedup of GASAL2 over SeqAn and Parasail is around 8x and 20X for all datasets.

#### Semi-global and global alignment

**Fig. 10:**
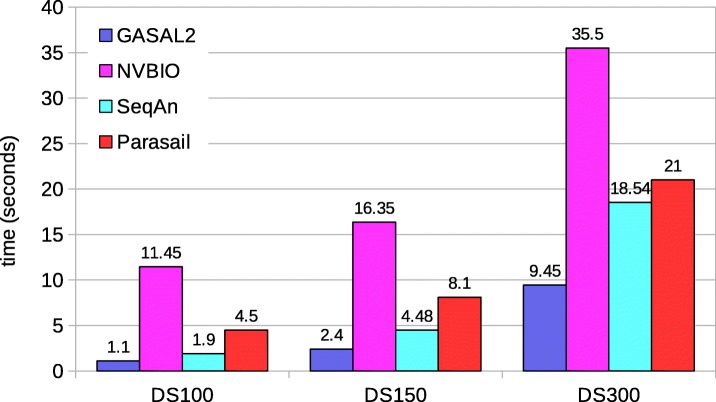
Total execution times for semi-global alignment computing only the score and end-position. The execution time of CPU-based libraries is obtained with 56 threads

There are many types of semi-global alignments. All types of semi-global alignments are possible with GASAL2. SeqAn supports all types of semi-global alignments. Prasail support 10 types. NVBIO supports only one type. In the paper we are showing the results for semi-global alignment supported by all the libraries i.e. gaps at end and beginning of the read sequence are not penalized. The relative performance of GASAL2, Parasail and SeqAn for the remaining types is similar. Figure 10 shows the total execution time of semi-global alignment computing only the score and end-position. Like local alignment, SeqAn only reports the alignment score. Whereas, GASAL2, Prasail and NVBIO compute the alignment score as well as the end-position of the alignment. The execution times for SeqAn and Parasail are obtained with 56 CPU threads. GASAL2 is 4x, 10x and 1.7x faster than Parasail, NVBIO and SeqAn, respectively for DS100. For DS150 the speedup of GASAL2 over Parasail, NVBIO and SeqAn is 3.4x, 6.8x and 1.9x, respectively. In case of DS300 GASAL2 is 2.2x, 3.75x and 2x faster than Parasail, NVBIO and SeqAn, respectively. The reasons for decreasing speedup over Parasail and NVBIO with increasing read lengths are the same as described for local alignment.

**Fig. 11:**
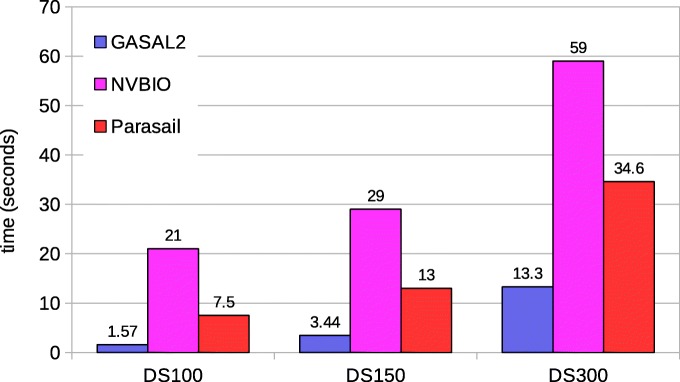
Total execution times for semi-global alignment computing start-position without traceback. The execution time of CPU-based libraries is obtained with 56 threads

Figure 11 shows the total execution time of the semi-global alignment computing start-position without traceback. SeqAn does not compute the start-position, which is hence omitted in the comparison. The results for Parasail are obtained with 56 CPU threads. The figure shows that GASAL2 is 4.7x, 3.7x and 2.6x faster than Parasail and 13x, 8.4x and 4.4x faster than NVBIO for DS100, DS150 and DS300, respectively.

**Fig. 12:**
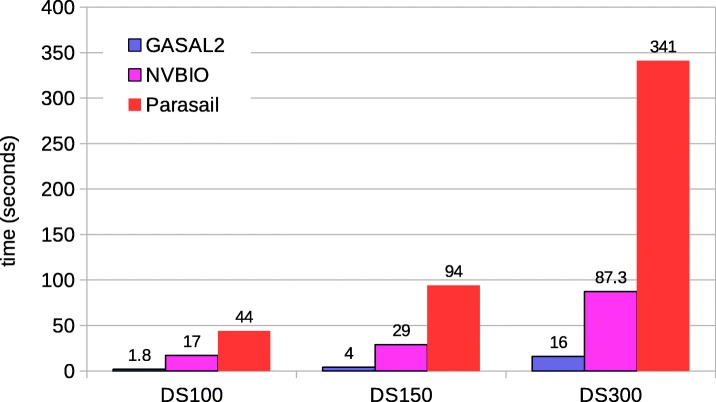
Total execution times for semi-global alignment with traceback computation. The execution time of CPU-based libraries is obtained with 56 threads except of SeqAn. For SeqAn the DS100 results are with 56 threads, whereas the DS150 and DS300 results are with 28 threads

Figure 12 shows the total execution of the semi-global alignment with traceback. The speedups of GASAL2 over NVBIO and Parasail (56 CPU threads) are similar to local alignment. For SeqAn the fastest execution time for DS100 is obtained with 56 threads, whereas for DS150 and DS300 28 threads are faster than 56 threads. GASAL2 is 3x, 3.5x and 13.5x faster than SeqAn for DS100, DS150 and DS300 respectively.

**Fig. 13:**
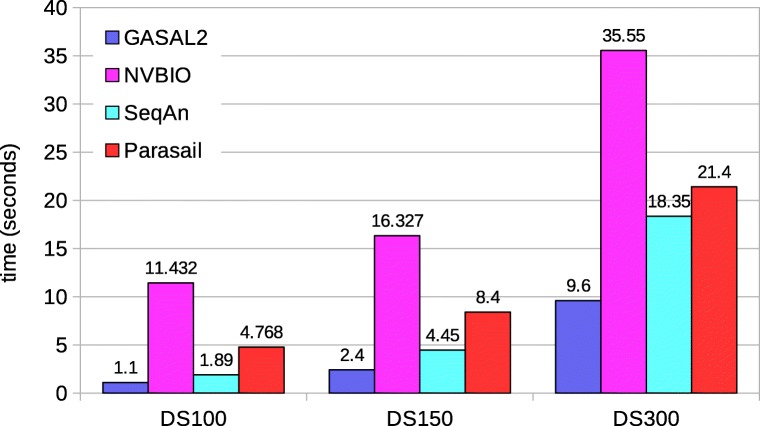
Total execution times for global alignment without traceback. The execution time of CPU-based libraries is obtained with 56 threads

**Fig. 14:**
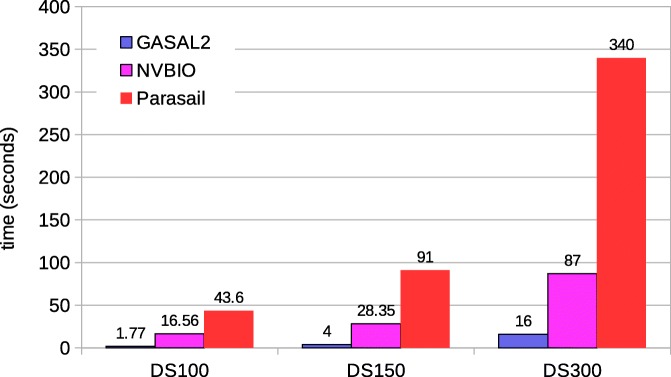
Total execution times for global alignment with traceback computation. The execution time of CPU-based libraries is obtained with 56 threads except for SeqAn. For SeqAn the DS100 results are with 56 threads, whereas the DS150 and DS300 results are with 28 threads

Figure 13 and 14 shows the total execution time required for global alignment without and with traceback, respectively. The thread settings and the speedups achieved by GASAL2 are similar to that of semi-global alignment. With traceback computation GASAL2 becomes even more faster than other CPU libraries. For semi-global and global alignments with traceback the speedup of GASAL2 over SeqAn increases with increasing read lengths.

## Discussion

GASAL2 is a GPU accelerated sequence alignment library. It can perform global alignment, local alignment and all types of semi-global alignment with and without traceback. It returns the alignment score, end-position and optionally the start-position of the alignment. It can also compute the second best local alignment score. Results show that GASAL2 is faster than NVBIO and state-of-the-art CPU-based SIMD libraries, making it a good choice for sequence alignment in high-throughput NGS data processing libraries. In the following, we show how to use the library to accelerate the BWA-MEM application.

### Case Study:

BWA-MEM is a well known *seed-and-extend* DNA read mapper. In the seeding step, it finds substrtings of the read that match exactly somewhere in the reference genome. In the extension step, BWA-MEM tries to align the whole read around that match. The algorithm used in the extension step is similar to local alignment, where the start-position is also calculated. We accelerated BWA-MEM using GASAL2. Two paired-end read datasets of length 150 bp (SRR949537) and 250 bp (SRR835433) are used. The experiments are run on an NVIDIA Tesla K40c GPU. The GPU host machine has two 2.4GHz Intel Xeon E5-2620 v3 processors and 32 gigabytes of RAM. Each processor has six cores with 2-way hyper-threading. The BWA-MEM version used in this case study is 0.7.13. We also accelerated BWA-MEM using GASAL and compared it with the results obtained with GASAL2. The original GASAL published in [22] has two shortcomings described in “GASAL2 versus GASAL and NVBIO” section: a) GASAL treats base ’N’ as an ordinary base. This causes BWA-MEM to abort due to an error. We updated GASAL so that it treats base ’N’ in the same manner as GASAL2, b) GASAL allocates and de-allocates the GPU memory just before and after the memory transfers between CPU and GPU, respectively. This causes the whole BWA-MEM application to slow down substantially due to repetitive GPU memory allocations and de-allocations. We updated GASAL so that the memory allocation and de-allocation are performed same as in GASAL2 i.e. only once, at the beginning and end of the application. The accelerated BWA-MEM is executed in the same manner as the original BWA-MEM (same command line arguments). The only difference between the accelerated BWA-MEM and the original version is that the seed-extension is performed on the GPU instead of CPU.

#### Execution timeline

**Fig. 15:**
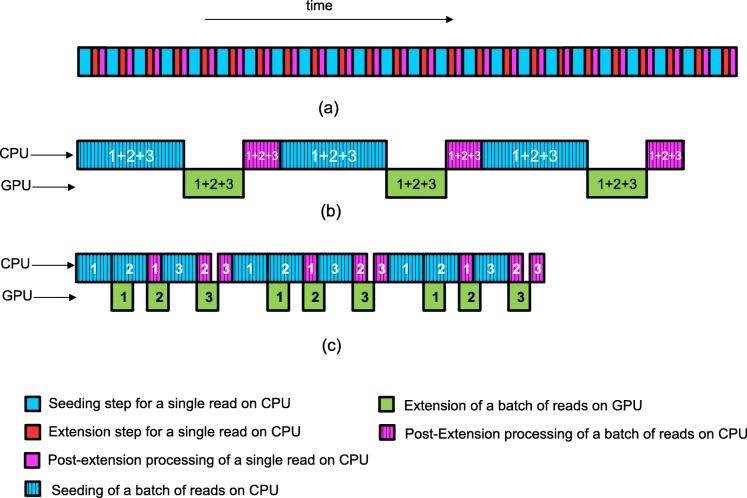
Execution timeline of original and accelerated BWA-MEM

Figure 15 shows the execution timeline of BWA-MEM before and after acceleration. Figure 15a shows the execution in the original BWA-MEM. Figure 15b shows the BWA-MEM execution with the extension step accelerated using GASAL. Note that the seeding and extension steps are performed for a batch of reads to mitigate the CPU-GPU memory transfer overhead and to fully utilize GPU resources. Furthermore, the thread running on the CPU remains idle while the extension is performed on the GPU. Figure 15c shows how the GASAL2 alignment function can be used for overlapping CPU and GPU execution. A batch of reads is further broken down into *sub-batches*, numbered 1, 2 and 3. CPU execution is overlapped with the seed extension on GPU. This is achieved via the GASAL2 asynchrnous alignment function call facility. Empty time slots on the CPU timeline are also present in (c), but these are much smaller than (b). These empty slots in (c) will not be present if extension on GPU is faster than post-extension processing or vice-versa. We test both approaches i.e. (b) and (c), to accelerate the extension step of BWA-MEM. In practice, due to load balancing (explained below) we used a batch size that varies between 5000 to 800 reads. The number of sub-batches are either 5 or 4.

#### Load balancing

In the original BWA-MEM, each thread is assigned a number of reads to process and one read is processed by a thread at a time. If a thread has finished processing all of its allocated reads, it will process the remaining reads of unfinished threads. Due to this, all of the threads remain busy until the whole data is processed resulting in maximum CPU utilization. On the other hand, in case of GPU acceleration reads are processed in the form of batches. Therefore, some threads may finish earlier than others and remain idle while waiting for all of the threads to finish. The idle time of these threads causes underutilization of the CPU. Decreasing the batch size helps to increase the CPU utilization, but at the same time may reduce the alignment function speedup due to increased data transfer overhead and poor GPU utilization. To circumvent this problem, we used dynamic batch sizes in our implementation. At the start, the batch size for each CPU thread is 5000 reads, but can be reduced to as low as 800 reads, depending upon the number of free threads which have finished processing there allocated reads. Doing so help to reduce the time wasted by a CPU thread in waiting for other threads to finish. We measured the *wasted time* as the difference between the finishing times of slowest and the fastest thread. By applying our dynamic batch size approach the wasted time is reduced by 3x for 150bp reads and 2x for 250 bp reads with 12 CPU threads.

#### Performance with 150bp reads

**Fig. 16:**
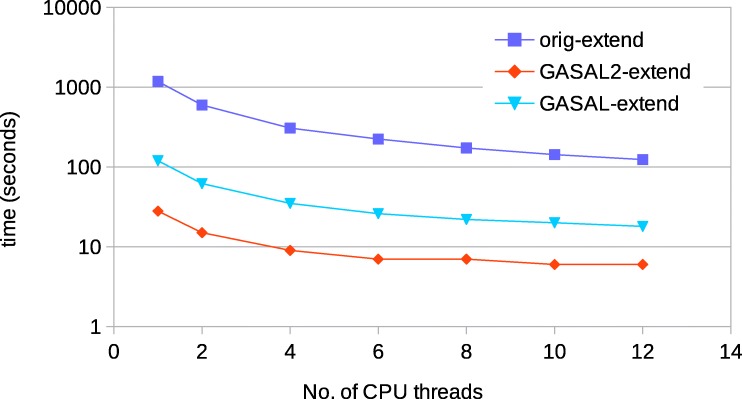
Time spent in the extension step of BWA-MEM for 150bp reads

For 150bp reads, Fig. 16 shows the comparison of time spent in the seed extension for the original BWA-MEM executed on the host CPU and the GPU accelerated BWA-MEM in which the seed extension is performed using GASAL2 alignment functions. The extension performed using GASAL2 (GASAL2-extend) is the sum of time to asynchronously call the GASAL2 alignment function and the time required in getting back the results using gasal_is_aln_async_done() function, in addition to the time of the empty slots before the post-processing of the last sub-batch. GASAL2-extend is more than 42x faster than the CPU time represented by original BWA-MEM extension function(orig-extend) for one thread, and over 20x faster for 12 CPU threads. Hence, the GASAL2 asynchronous alignment function allows to completely eliminate the seed extension time. The GASAL alignment function (GASAL-extend) is 3-4x slower than GASAL2-extend and is hence around 7-10x fassimilarter than orig-extend.

**Fig. 17:**
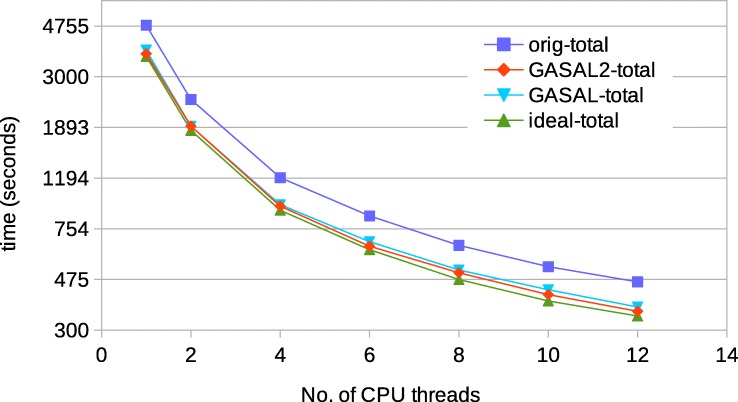
Total execution time of BWA-MEM for 150 bp reads

Figure 17 shows the total execution times of the original BWA-MEM and GASAL2 for 150bp data. The *ideal-total* is total execution time for the case in which the time spent in the extension step is zero, and thus, represents the maximum achievable speedup. For 1 to 4 CPU thread, the GPU speedup is almost identical to the ideal one. For higher CPU threads, the speedup is slightly smaller than ideal. For 12 threads, the GASAL2 speedup and ideal speedup are 1.3 and 1.36, respectively. Since the time consumed by the seed extension function in BWA-MEM is 25-27%, the total execution time of GASAL is only slightly higher than GASAL2. For 12 threads, the GASAL speedup is 1.26. The main cause of the difference between ideal and actual speedup for higher number of CPU threads is imperfect load balancing between the CPU threads.

#### Performance with 250 bp reads

**Fig. 18:**
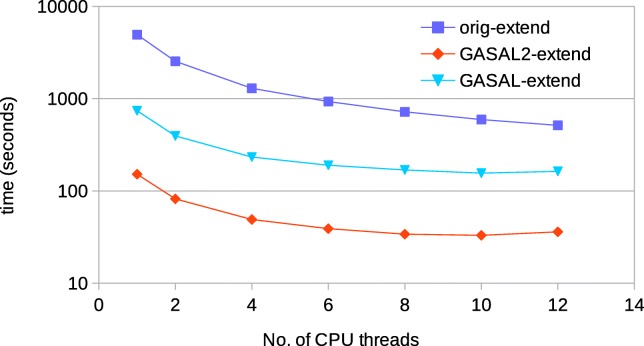
Time spent in the extension step of BWA-MEM for 250bp reads

Same analysis is repeated for 250 bp reads. Figure 18 shows the seed extension time of original BWA-MEM and GASAL2 alignment functions. GASAL2-extend is 32x to 14x faster than orig-extend for 1 to 12 CPU threads, respectively. The reduction in speed-up as compared to 150bp reads is due to reduction in GPU alignment kernel speed for longer reads, which widens the empty slots in the CPU timeline of Fig. 15c. GASAL-extend is 7x to 3x faster than CPU extension for 1 to 12 CPU threads, respectively. This means that GASAL-extend is 4-5x slower than GASAL2-extend. Hence, for longer reads the speedup of GASAL2 over GASAL increases.

**Fig. 19:**
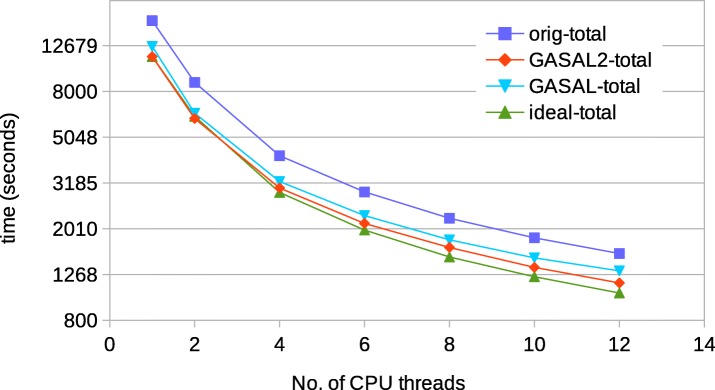
Total execution time of BWA-MEM for 250 bp reads

Figure 19 shows the total execution time for 250 bp reads. For up to 2 CPU threads, GASAL2-total, GASAL-total and ideal-total all are the same. Above 2 CPU threads, GASAL2-total becomes faster than GASAL-total. For 12 CPU threads, the ideal speedup is 1.49 whereas the speedup with GASAL2 and GASAL is 1.35 and 1.2, respectively. The gap between the ideal speedup and speedup achieved with GASAL2 is larger for 250 bp reads as compared to 150 bp reads. This happened due to imperfect load balancing between threads as well as decreased speedup of the seed extension step for 250bp reads.

In summary GASAL2 gives seed-extension speedup in excess of 10x even when 12 CPU threads share a single NVIDIA Tesla K40c GPU.

## Conclusions

In this paper, we presented GASAL2, a high performance and GPU accelerated library, for pairwise sequence alignment of DNA and RNA sequences. The GASAL2 library provides accelerated kernels for local, global as well as semi-global alignment, allowing the computation of the alignment with and without traceback. It can also compute the start position without traceback. In addition, one-to-one as well as all-to-all and one-to-many pairwise alignments can be performed. GASAL2 uses the novel approach of also performing the sequence packing on GPU, which is over 750x faster than the NVBIO approach. GASAL2 alignment functions are asynchronous/non-blocking which allow fully overlapping CPU and GPU execution. GASAL2 can compute all types of semi-global alignments. These represent unique capabilities not available in any earlier GPU sequence alignment library. The paper compared GASAL2’s performance with the fastest CPU-optimized SIMD implementations such as SeqAn, ksw, Parasail and NVBIO (NVIDIA’s own GPU library for sequence analysis of high-throughput sequencing data). Experimental results performed on the Geforce GTX 1080 Ti GPU show that GASAL2 is up to 5.35x faster than 56 Intel Xeon threads and up to 10x faster than NVBIO with a read length of 100bp, computing only the score and end-position. For 150bp reads, the speedup of GASAL2 over CPU implementations (56 Intel Xeon threads) and NVBIO is up to 4.75x and up to 7x, respectively. With 300bp reads, GASAL2 is up to 3.4x faster than CPU (56 Intel Xeon threads) and NVBIO. The speedup of GASAL2 over CPU implementations (56 Intel Xeon threads) computing start-position without traceback is up to 6x, 5.3x and 4x for 100, 150 and 300bp reads, respectively. With start-position computation, the speedup of GASAL2 over NVBIO is up to 13x, 8.7x and 4.4x for 100, 150 and 300bp reads, respectively. With traceback computation GASAL2 becomes even faster. GASAL2 traceback alignment is 13x and 20x faster than SeqAn and Parasail for read lengths of up to 300 bases. The GPU traceback alignment kernel of GASAL2 is 4x faster than NVBIO’s kernel, giving an overall speedup of 9x, 7x and 5x for 100, 150 and 300bp reads, respectively. GASAL2 is used to accelerate the seed extension function of BWA-MEM DNA read mapper. It is more than 20x faster than the CPU seed extension functions with 12 CPU threads. This allows us to achieve nearly ideal speedup for 150 bp reads. The library provides easy to use APIs to allow integration into various bioinformatics tools. GASAL2 is publicly available and can be downloaded from: https://github.com/nahmedraja/GASAL2.

## Availability and requirements

**Project name:** GASAL2- GPU Accelerated Sequence Alignment Library.

**Project home page:** https://github.com/nahmedraja/GASAL2

**Operating system(s):** Linux

**Programming language:** C++, CUDA

**Other requirements:** CUDA toolkit version 8 or higher.

**License:** Apache 2.0

**Any restrictions to use by non-academics:** Not applicable

## Data Availability

Not applicable.

## Abbreviations

AVX2: Advanced vector extensions version-2
CPU: Central processing unit
CUDA: Compute unified device architecture
GPU: Graphical processing unit
NGS: Next generation sequencing
SIMD: Single instruction multiple data
SM: Streaming multiprocessor
SP: Streaming processor
SSE: Streaming SIMD extensions

## References

[1] Li H. Aligning sequence reads, clone sequences and assembly contigs with BWA-MEM. arXiv. 2013.

[2] Langmead B, S S (2012). Fast gapped-read alignment with Bowtie 2. Nat Methods.

[3] Huang X, Yang S-P. Generating a Genome Assembly with PCAP. 2002. 10.1002/0471250953.bi1103s11 18428744

[4] de la Bastide M, McCombie WR. Assembling Genomic DNA Sequences with PHRAP. 2002. 10.1002/0471250953.bi1104s17 18428783

[5] Salmela L, Schröder J (2011). Correcting errors in short reads by multiple alignments. Bioinformatics.

[6] Kao W-C, Chan AH, Song YS (2011). ECHO: a reference-free short-read error correction algorithm. Genome Res.

[7] Poplin R, et al. Scaling accurate genetic variant discovery to tens of thousands of samples. bioRxiv. 2017.

[8] Needleman SB, Wunsch CD (1970). A general method applicable to the search for similarities in the amino acid sequence of two proteins. J Mole Biol.

[9] Smith TF, Waterman MS (1981). Identification of common molecular subsequences. J Mole Biol.

[10] Gotoh O (1982). An improved algorithm for matching biological sequences. J Mole Biol.

[11] Alexandrov VN, van Albada GD, Sloot PMA, Dongarra J (2006). GPU Accelerated Smith-Waterman.

[12] Liu Y, Wirawan A, Schmidt B (2013). CUDASW++ 3.0: Accelerating Smith-Waterman protein database search by coupling CPU and GPU SIMD instructions. BMC Bioinformatics.

[13] Hasan L, Kentie M, Al-Ars Z (2011). DOPA: GPU-based protein alignment using database and memory access optimizations. BMC Res Notes.

[14] Ren S, Bertels K, Al-Ars Z (2018). Efficient Acceleration of the Pair-HMMs Forward Algorithm for GATK HaplotypeCaller on Graphics Processing Units. Evol Bioinforma.

[15] Ren S, Ahmed N, Bertels K, Al-Ars Z. An Efficient GPU-Based de Bruijn Graph Construction Algorithm for Micro-Assembly. In: 2018 IEEE 18th International Conference on Bioinformatics and Bioengineering (BIBE): 2018. p. 67–72.

[16] Kalaiselvi T, Sriramakrishnan P, Somasundaram K (2017). Survey of using gpu cuda programming model in medical image analysis. Informa Med Unlocked.

[17] Sriramakrishnan P, Kalaiselvi T, Rajeswaran R (2019). Modified local ternary patterns technique for brain tumour segmentation and volume estimation from mri multi-sequence scans with gpu cuda machine. Biocyber Biomed Eng.

[18] Bhosale P, Staring M, Al-Ars Z, Berendsen FF. GPU-based stochastic-gradient optimization for non-rigid medical image registration in time-critical applications. In: SPIE Medical Imaging 2018: 2018.

[19] Blazewicz J, Frohmberg W, Kierzynka M, Pesch E, Wojciechowski P (2011). Protein alignment algorithms with an efficient backtracking routine on multiple GPUs. BMC Bioinformatics.

[20] Liu Y, Schmidt B (2015). GSWABE: faster GPU-accelerated sequence alignment with optimal alignment retrieval for short DNA sequences. Concurr Comput: Pract Exp.

[21] Pantaleoni J, Subtil N. NVBIO. 2015. https://nvlabs.github.io/nvbio. Accessed 1 October, 2017.

[22] Ahmed N, Mushtaq H, Bertels K, Al-Ars Z. GPU accelerated API for alignment of genomics sequencing data. In: 2017 IEEE International Conference on Bioinformatics and Biomedicine (BIBM): 2017. p. 510–5.

[23] Li H. wgsim: Reads simulator. https://github.com/lh3/wgsim. Accessed 1 October, 2017.

[24] Ehrhardt M, Rahn R, Reinert K, Budach S, Costanza P, Hancox J (2018). Generic accelerated sequence alignment in SeqAn using vectorization and multi-threading. Bioinformatics.

[25] R R. DP Bench - A benchmark tool for SeqAn’s alignment engine.

[26] Chaos A. Klib: a Generic Library in C. https://github.com/attractivechaos/klib. Accessed 2 January, 2019.

[27] Zhao Mengyao, Lee Wan-Ping, Garrison Erik P., Marth Gabor T. (2013). SSW Library: An SIMD Smith-Waterman C/C++ Library for Use in Genomic Applications. PLoS ONE.

[28] Daily J (2016). Parasail: SIMD C library for global, semi-global, and local pairwise sequence alignments. BMC Bioinformatics.

[29] Šošić M, Šikić M (2017). Edlib: a C/C++ library for fast, exact sequence alignment using edit distance. Bioinformatics.

[30] Benson G, Loving J, Hernandez Y (2014). BitPAl: a bit-parallel, general integer-scoring sequence alignment algorithm. Bioinformatics.

[31] Lan H, Zhang J, Chan Y, Liu W, Shang Y, Schmidt B. BGSA: a bit-parallel global sequence alignment toolkit for multi-core and many-core architectures. 2018. 10.1093/bioinformatics/bty930 30445566

[32] Myers EW, Miller W (1988). Optimal alignments in linear space. Bioinformatics.

