# Correction to: GASAL2: a GPU accelerated sequence alignment library for high-throughput NGS data

**DOI:** 10.1186/s12859-019-3185-7

**Published:** 2019-11-19

**Authors:** Nauman Ahmed, Jonathan Lévy, Shanshan Ren, Hamid Mushtaq, Koen Bertels, Zaid Al-Ars

**Affiliations:** 1 Delft University of Technology, Delft, Netherlands and University of Engineering and Technology, Lahore, Pakistan; 2 Delft University of Technology, Netherlands, Delft, Netherlands; 3 Maastricht UMC+, Netherlands, Maastricht, Netherlands

**Correction to: BMC Bioinformatics (2019) 20:520**

**https://doi.org/10.1186/s12859-019-3086-9**

Following publication of the original article [1], the author requested changes to the Figs. 4, 7, 8, 9, 12 and 14 to align these with the text. The corrected figures are supplied below.

**Fig. 4:**
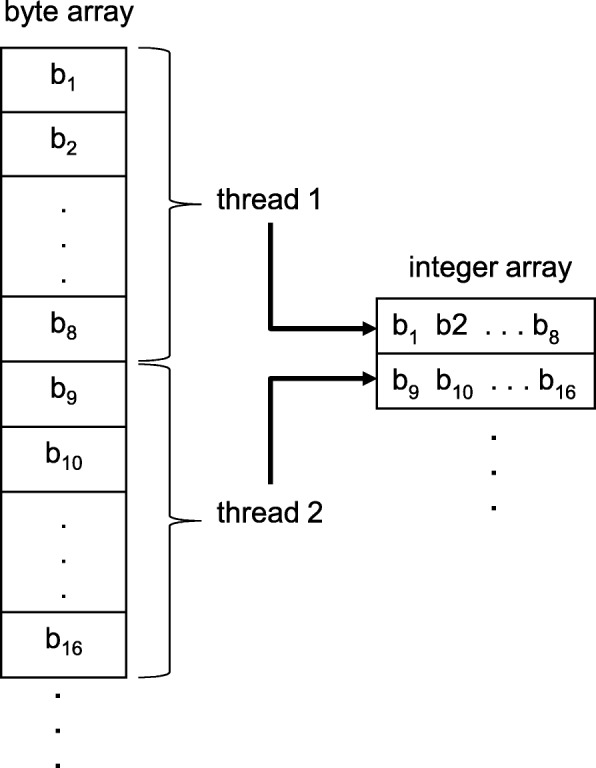
Packing the sequences on GPU. *b*_1_,*b*_2_,…, are the bases

**Fig. 7:**
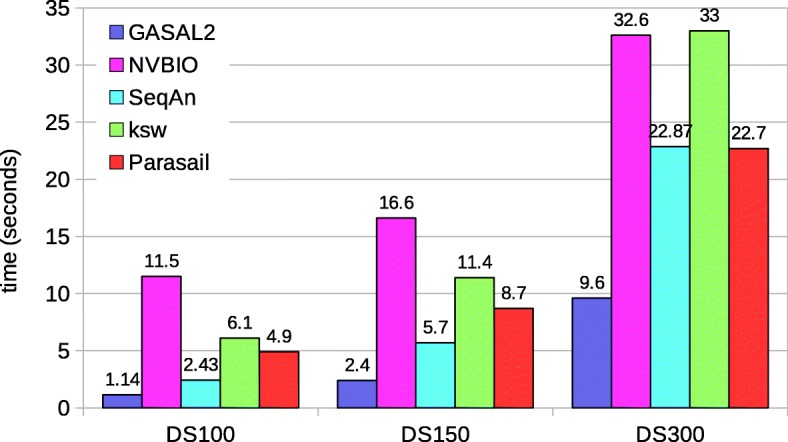
Total execution times for local alignment computing only the score and end-position. The execution time of CPU-based libraries is obtained with 56 threads

**Fig. 8:**
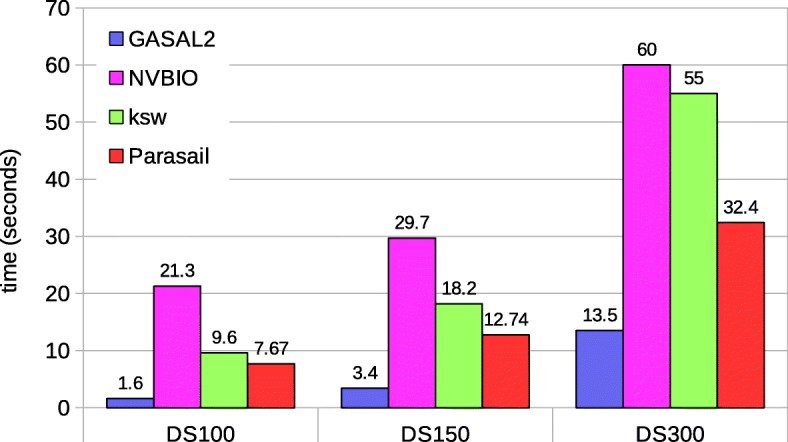
Total execution times for local alignment computing start-position without traceback. The execution time of CPU-based libraries is obtained with 56 threads

**Fig. 9:**
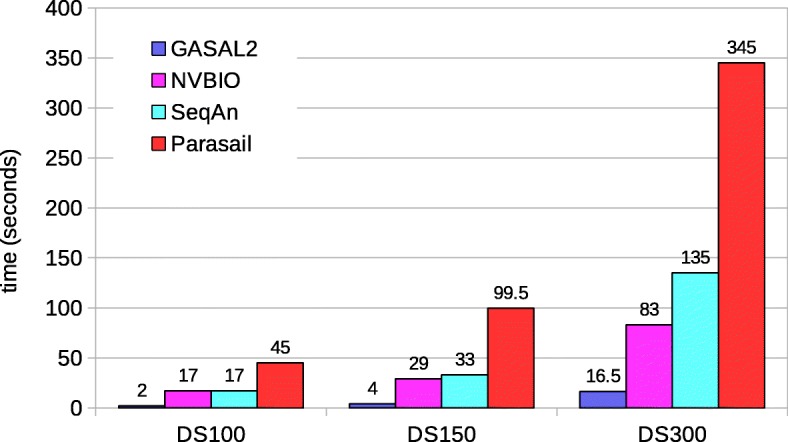
Total execution times for local alignment with traceback computation. The execution time of CPU-based libraries is obtained with 56 threads

**Fig. 12:**
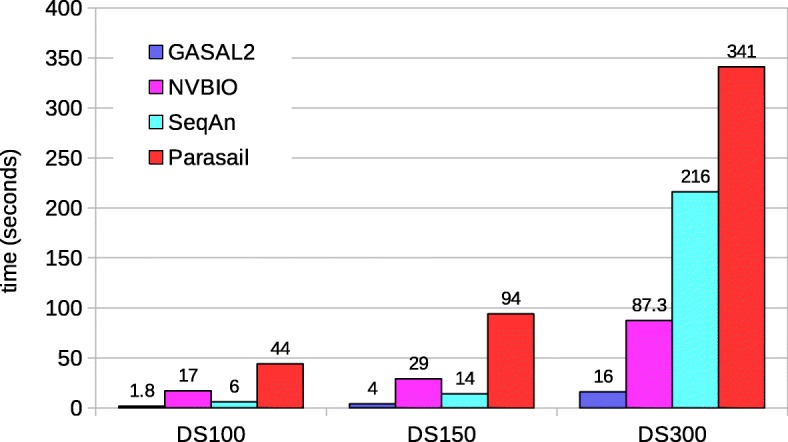
Total execution times for semi-global alignment with traceback computation. The execution time of CPU-based libraries is obtained with 56 threads except of SeqAn. For SeqAn the DS100 results are with 56 threads, whereas the DS150 and DS300 results are with 28 threads

**Fig. 14:**
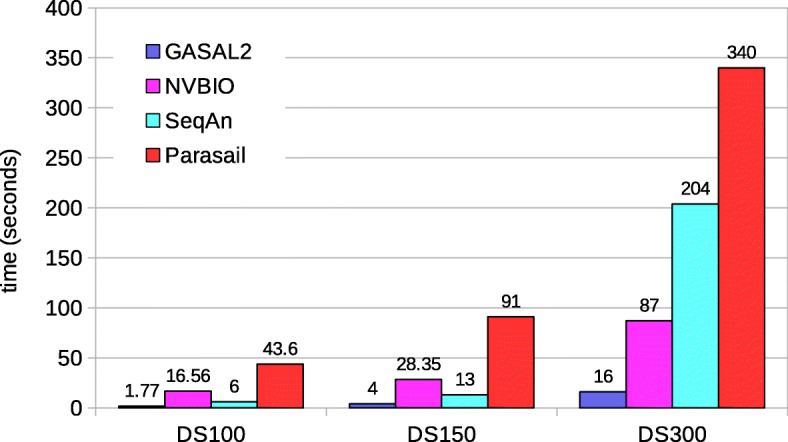
Total execution times for global alignment with traceback computation. The execution time of CPU-based libraries is obtained with 56 threads except for SeqAn. For SeqAn the DS100 results are with 56 threads, whereas the DS150 and DS300 results are with 28 threads

The original article [1] has been corrected.

[Typesetter, please insert new supplied figure in package]
